# CCR1 and CCR2 Coexpression on Monocytes Is Nonredundant and Delineates a Distinct Monocyte Subpopulation

**DOI:** 10.4049/jimmunol.2400007

**Published:** 2024-06-03

**Authors:** Laura Medina-Ruiz, Robin Bartolini, Heather Mathie, Heba A. Halawa, Madeleine Cunningham, Gerard J. Graham

**Affiliations:** Chemokine Research Group, Centre for Immunobiology, School of Infection and Immunity, College of Medical, Veterinary and Life Sciences, University of Glasgow, Glasgow, United Kingdom

## Abstract

The interactions between chemokines and their receptors, particularly in the context of inflammation, are complex, with individual receptors binding multiple ligands and individual ligands interacting with multiple receptors. In addition, there are numerous reports of simultaneous coexpression of multiple inflammatory chemokine receptors on individual inflammatory leukocyte subtypes. Overall, this has previously been interpreted as redundancy and proposed as a protective mechanism to ensure that the inflammatory response is robust. By contrast, we have hypothesized that the system is not redundant but exquisitely subtle. Our interests relate to the receptors CCR1, CCR2, CCR3, and CCR5, which, together, regulate nonneutrophilic myeloid cell recruitment to inflammatory sites. In this study, we demonstrate that although most murine monocytes exclusively express CCR2, there is a small subpopulation that is expanded during inflammation and coexpresses CCR1 and CCR2. Combinations of transcript and functional analysis demonstrate that this is not redundant expression and that coexpression of CCR1 and CCR2 marks a phenotypically distinct population of monocytes characterized by expression of genes otherwise typically associated with neutrophils. Single-cell RNA sequencing confirms this as a monodisperse population of atypical monocytes. This monocytic population has previously been described as having immunosuppressive activity. Overall, our data confirm combinatorial chemokine receptor expression by a subpopulation of monocytes but demonstrate that this is not redundant expression and marks a discrete monocytic population.

## Key Points

- CCR2+ve and CCR1/2+ve monocytes are transcriptionally distinct.
- CCR1/2+ve monocytes display a neutrophilic gene signature.

## Introduction

Leukocyte migration in vivo is regulated in the main by proteins called “chemokines,” which are defined on the basis of a conserved cysteine motif (1, 2). The broader chemokine family is also divided into four subfamilies according to the specific configuration of this cysteine motif, with these subfamilies comprising the CC, CXC, XC, and CX3C chemokines. All characterized chemokine receptors belong to the seven-transmembrane spanning family of G protein–coupled receptors, and currently 10 receptors for CC chemokines, 6 for CXC chemokines, and single receptors for the XC and CX3C chemokines have been identified (3). In addition, there is a small subfamily of atypical chemokine receptors that are preferentially expressed on stromal cells and that help to shape the chemokine-driven response in a range of tissue and cellular contexts (4, 5). Overall, therefore, chemokines and their receptors are the most prominent regulators of leukocyte migration in vivo under both inflammatory and homeostatic conditions (2, 6).

Understanding roles for chemokines and their receptors in inflammation is complicated by the fact that individual inflammatory chemokine receptors interact with multiple chemokines and are therefore “promiscuous.” In addition, the ligands can interact with multiple receptors, leading to confusing ligand–receptor interaction networks (7). In addition, there are numerous reports of coexpression of multiple inflammatory chemokine receptors, including receptors interacting with the same ligands, on individual inflammatory leukocyte subtypes (3, 8). Together these complexities have been interpreted as redundancy within the inflammatory chemokine system, which ensures molecular backups at all stages of the inflammatory response (6, 9–11).

We have been studying this issue of complexity and redundancy in the context of four inflammatory chemokine receptors that regulate nonneutrophilic myeloid cell mobilization, recruitment, and migration to inflamed sites. These receptors are CCR1, CCR2, CCR3, and CCR5 (inflammatory CCRs [iCCRs]), which together occupy a single tight chromosomal locus in the mammalian genome. We have deleted this entire locus (12) and also generated compound reporter mice (REP mice) in which expression of each of these receptors is marked by a spectrally distinct fluorescent reporter allowing tracking of each of the receptors on individual leukocytes at rest and during the inflammatory response (13). Overall, our results indicate a lack of redundancy in either the use or expression of the iCCRs in inflammation and suggest that the chemokine and chemokine receptor interaction network is more specific than previously realized. Recently, we demonstrated that murine inflammatory leukocytes do not generally express more than one of the iCCRs, refuting the notion of multiple and redundant receptor expression (13). However, we have identified an exception to this general rule. Although most inflammatory monocytes only express CCR2 from within the iCCR locus (CCR2+ve), we have identified a small subpopulation that coexpresses CCR2 and CCR1 (CCR1/2+ve) (13). This population is expanded during the inflammatory response (13).

The purpose of the present study was to compare CCR2+ve with CCR1/2+ve monocytes to determine whether they represent the same cell type, with redundant chemokine receptor expression, or whether they represent discrete cellular populations. In this study, we show that CCR1/2+ve monocytes are similar to but transcriptionally distinct from CCR2+ve monocytes and that the transcriptional differences between these two populations indicate that the CCR1/2+ve monocytes represent a previously reported monocytic population with neutrophilic gene expression. The CCR1/2+ve population remains nonredundantly reliant on CCR2 for mobilization from the bone marrow and recruitment to inflammatory sites. Overall, therefore, these results are not in keeping with the concept of chemokine receptor redundancy and highlight coexpression of CCR1 and CCR2 as a marker of a specific “neutrophilic” monocyte subpopulation.

## Materials and Methods

### Animals

Wild-type and REP (13) mice were housed in a specific pathogen-free animal facility at the University of Glasgow. All animal experimentation was carried out under the auspices of a U.K. Home Office license, and all procedures were approved by the local University of Glasgow ethics committee. All mice used were female and between the ages of 8 and 12 wk.

### Resting bone marrow isolation

Resting mice were euthanized and perfused with 20 ml PBS containing 2 mM EDTA (Thermo Fisher Scientific). Bone marrow was extracted from the femur and tibia; RBCs were lysed using an ACK lysis solution (Thermo Fisher Scientific) following the manufacturer’s instructions; and leukocytes were stained for flow cytometric analysis or for monocyte isolation and transcriptomic analysis.

### Air pouch model

Sterile air (3 ml) was injected s.c. into the mouse dorsum every 2 d on three occasions. One day after the final air injection, 1 ml autoclaved carrageenan (1% w/v in PBS; Sigma-Aldrich) was injected into the air pouch. Twenty-four hours later (and 12 h before cull), mice were injected i.v. with chemokines to enhance monocyte extravasation. Injections included 100 ml 0.1% BSA in PBS (vehicle control) and 100 ml CCL3 (2.5 mg) or 100 µl CCL7, CCL3, CCL5, and CXCL5 (2.5 mg each). After the cull, 3 ml buffer (PBS containing 1 mM EDTA and 1% w/v FBS; Sigma-Aldrich) was used to flush the air pouches, and the lavage fluid was collected. The membrane surrounding the air pouch was then dissected and digested for 1 h at 37°C with shaking at 800 rpm in 1 ml HBSS containing 0.44 Wünsch units of Liberase (Roche). Membrane cell suspensions were passed through 70-μm nylon mesh filters and washed. Blood, bone marrow, air pouch lavage fluid, and digested membrane samples were then analyzed for cellular content via flow cytometry.

### Implantation of cytokine-loaded osmotic pumps

Osmotic pumps (Alzet osmotic pumps, model 2001; Charles River) were loaded with a cytokine mixture containing IL-3 (15 ng/µl), IL-6 (16 ng/µl), GM-CSF (15 ng/µl), and IFN-α (2.083 ng/µl) or with vehicle PBS. Mice were anesthetized using inhaled isoflurane (2% isoflurane and 2 L O_2_/min) followed by a s.c. injection of carprofen (100 μl at 1 mg/ml) for analgesia. Then, a small cavity was generated under the dorsal skin, where the cytokine-loaded osmotic pump was inserted. Infusion of PBS or the cytokine mixture (15 ng IL-3/h, 16 ng IL-6/h, 15 ng GM-CSF/h, and 2.083 ng IFNα/h) was maintained for 7 d. After 7 d, animals were sacrificed and perfused, and bone marrow was extracted as detailed above. The membrane surrounding the osmotic pump was isolated and digested by shaking at 1000 rpm and 37°C in 1 ml HBSS (Thermo Fisher Scientific) containing 0.44 Wünsch units of Liberase (Roche) for 1 h. After digestion, Liberase was neutralized with 20 μl FBS, and cell suspensions were filtered through 70-μm nylon mesh membranes, washed with PBS, and stained for flow cytometric analysis.

### Flow cytometry and monocyte sorting

Cell suspensions were stained for 20 min at 4°C with 100 μl fixable viability stain (eBioscience) and washed in FACS buffer (PBS containing 2 mM EDTA and 2% FBS). Next, cells were stained for 20 min at 4°C with 50 μl subset-specific Ab cocktails (Supplemental Table I) and washed in FACS buffer. For flow cytometric analysis, stained cells were fixed for 20 min at 4°C in 100 μl fixation buffer (BioLegend) and analyzed on a BD LSRFortessa flow cytometer (BD Biosciences). For monocyte isolation and transcriptomic analysis, stained cells (Supplemental Table II) were analyzed on a FACSAria sorter (BD Biosciences) without previous fixation. Monocytes expressing either CCR1/2 or CCR2 only were sorted in RLT buffer (Qiagen) containing 10 μl/ml of 2-ME and stored at −80°C for RNA extraction.

### Macrophage culture

CCR2 only (mRuby2+) and CCR1/CCR2 coexpressing (mRuby2+/Clover+) bone marrow inflammatory monocytes were sorted from resting REP mice (CD11b^+^ Ly6C^++^ Ly6G^−^ SiglecF^−^) on a FACSAria sorter (BD Biosciences) and cultured in 12-well plates for 5 d at a starting concentration of 150,000 cells/well in 2 ml L929 conditioned media (Glasgow’s MEM, 15% L929 conditioned media, 10% FBS, l-glutamine, 50 μM 2-MW, and Primocin) (14). Medium (1 ml) was replaced at day 3. After 5 d, macrophages (CD11b^+^ F480^+^) and CD11b^+^F480^−^ cells were detached with TrypLe Select (A12177.01, Life Technologies) and analyzed via flow cytometry for surface markers and fluorescent reporter protein expression (CCR1 = Clover, CCR2 = mRuby2, CCR3 = mTagBFP2, CCR5 = IRFP682).

### RNA isolation and bulk RNA sequencing (RNA-seq)

RNA from sorted monocytes was isolated using the RNeasy Micro Kit (Qiagen) as per the manufacturer’s instructions. Next, mRNA libraries were prepared using the NEBNext Single Cell/Low Input RNA Library Prep Kit for Illumina (New England Biolabs). Finally, paired-end sequencing was performed in a NextSeq2000 sequencing platform (Illumina) aiming for 40 million reads sequencing depth. Bulk RNA-Seq datasets were subject to the following pipeline. First, fastQ files were assessed using FastP (15), and then they were aligned to the mouse reference genome (GRCm38.91) using STAR (2.7.10a) (16) with –quantMode GeneCounts, –outFilterMultimapNmax 1, and –outFilterMatchNmin 35. We used a Star index with a –sjdbOverhang of the maximum read length −1. Read count files were merged, and genes with a mean of less than one read per sample were excluded from further analysis. The expression and differential expression values were generated using DESeq2 (version 1.24) (17). For differential comparisons, we used an A versus B model with no additional covariates. All other parameters were left to default. The processed data were then visualized using Searchlight (18), specifying one differential expression workflow for each comparison, an absolute log_2_ fold cutoff of 1, and adjusted *p* value of 0.05. For overrepresentation analysis, we used the STRING 11.5 database, with significance set to <0.05. All other parameters were left to default.

### Single-cell RNA-seq library preparation and sequencing analysis

CCR1/2+ monocytes (CD45^+^, CD11b^+^, Ly6C^hi^, Ly6G^−^, SiglecF^−^, CCR2^+^, CCR1^+^) were sorted from the bone marrow of REP mice using the BD FACSAria and collected into 1% BSA in PBS. Single-cell libraries were generated using the 10X Genomics Chromium NextGEM Single Cell 3′ kit (version 3.1) and following the manufacturer’s instructions. Cells were added to the 10X Genomics NextGEM Chip G at a concentration between 700 and 1200 cells/µl, with a targeted cell recovery of 7000 cells. Cells were combined with Gel Beads-in-Emulsion on the Chromium Controller prior to lysis and reverse transcription. Gel Beads-in-Emulsion were then broken, and cDNA amplification and fragmentation were performed. After fragmentation, the i7 sample index was ligated, and Illumina P5 and P7 adapters were added. Sequencing was performed by Glasgow Polyomics on the Illumina NextSeq 2000 sequencer using a custom paired-end sequencing run (28 × 90 bp) to yield 250 million reads. Reads were aligned using the count function in 10X Genomics Cell Ranger, and outputs were imported into R (version 4.2.2) for downstream analysis using Seurat (19, 20). Cells were removed from the analysis if they failed quality control thresholds for the number of features and counts; the range of features included in downstream analysis was 200–3,200, and the range of counts was 100–20,000. In addition, cells were removed if they did not meet the threshold for mitochondrial percentage (<2.5%). Data were normalized, and principal component analysis was performed using the function RunPCA. Eleven principal components were included in downstream analysis. The FindClusters function was run at a range of resolutions (0.5–0.1) to select a resolution that could discriminate unique subpopulations based on Top20 gene expression; a resolution of 0.1 was selected. Fifty-nine contaminating B cells were subsetted out of the data for downstream analysis. Data were rescaled, and cell cycle analysis was performed using the function CellCycleScoring. The effect of the cell cycle was then regressed out of the data. Uniform Manifold Approximation and Projection (UMAP) dimensionality reduction was applied for visualization.

### Statistics

All statistical tests were performed using GraphPad Prism software.

### Data availability

The bulk RNA-seq and singe-cell RNA-seq data have been deposited in the Gene Expression Omnibus (GSE251648).

## Results

### CCR1/2 expression marks a distinct population of bone marrow monocytes

**FIGURE 1.**
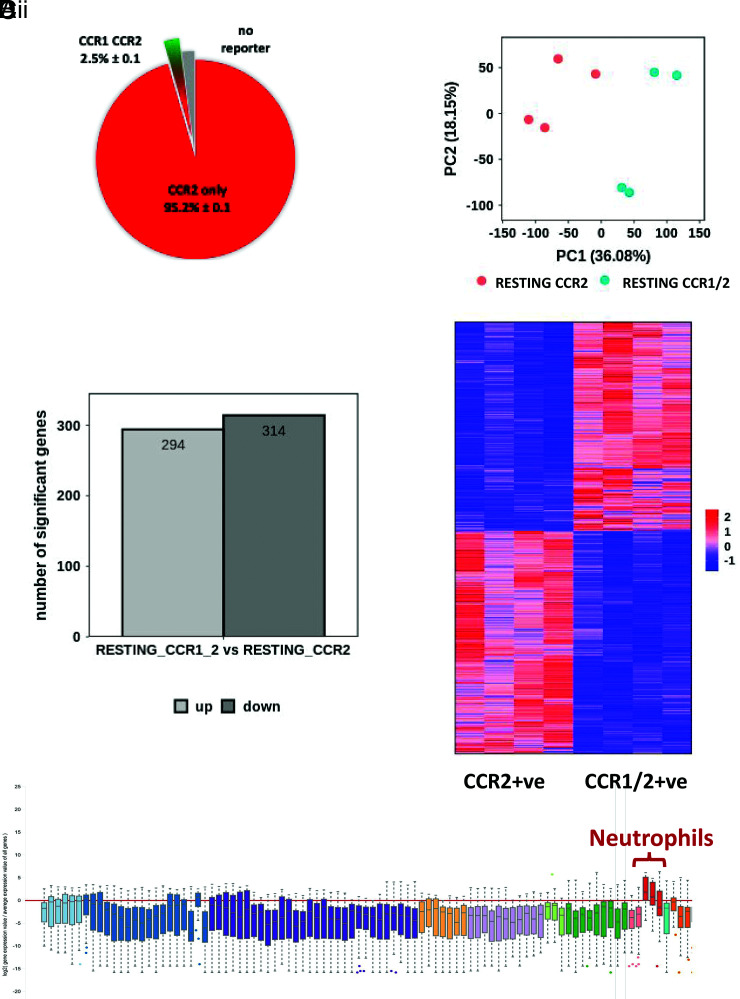
CCR2+ve and CCR1/2+ve monocytes are transcriptionally distinct. (**A**) Pie chart showing the proportion of CCR1/2+ve monocytes (green/red) and CCR2+ve monocytes (red) within the total bone marrow monocyte population. These data were obtained from flow cytometric analysis of REP mouse peripheral blood monocytes. (**B**) Principal component analysis of bulk RNA-seq data from bone marrow–derived CCR1/2+ve monocytes and CCR2+ve monocytes. (**C**) (i) Bar graph demonstrating the number of up- and downregulated genes expressed by CCR1/2+ve monocytes versus CCR2+ve monocytes. (ii) Heatmap demonstrating distinct gene expression patterns in CCR1/2+ve monocytes and CCR2+ve monocytes. (**D**) Box plot showing association of CCR1/2+ve monocytes with a neutrophilic transcription profile. This was obtained by entering the top 200 genes, preferentially expressed in CCR1/2+ve monocytes, into the engine server using the MyGeneset program, which analyzes transcript expression across immune and stromal cell populations.

In terms of iCCRs, and as previously reported, although the majority of monocytes in the bone marrow and blood only express CCR2, there is a subpopulation that coexpresses CCR1 and CCR2 (Supplemental Fig. 1A, 1B). This population is also present, and indeed slightly expanded, in the spleen (Supplemental Fig. 1C). To determine whether CCR1/2+ve and CCR2+ve monocytes represent variations in chemokine receptor expression within an otherwise homogeneous monocyte population or are distinct populations, we isolated CCR1/2+ve and CCR2+ve monocytes from REP mouse (13) bone marrow (Fig. 1A). Bulk transcriptomic analysis was then carried out, and principal component analysis (Fig. 1B) indicated that these populations are closely related but distinct, with ∼300 upregulated and 300 downregulated genes separating the populations (Fig. 1Ci, 1Cii). Both populations displayed an essentially monocytic core gene signature. However, gene ontology analysis (Supplemental Fig. 2A) indicated that transcripts upregulated in CCR2+ve monocytes included genes involved in the regulation of hemopoiesis along with IFN-γ responses and cell adhesion. In contrast, transcripts upregulated in CCR1/2+ve monocytes (Supplemental Fig. 2B) were indicative of active cell division, suggesting a more proliferatively active cellular population.

Analysis of the immune/inflammatory lineage affiliation of the differentially expressed genes in these two populations (using ImmGen) failed to reveal any specific lineage association of the transcripts preferentially expressed in CCR2+ve monocytes, which were broadly distributed across the tested populations (not shown). Strikingly, however, transcripts preferentially expressed in CCR1/2+ve monocytes displayed a preferential association with a neutrophilic transcription profile (Fig. 1D). Many of these transcripts (see Table I) encode typical components of neutrophil granules, but the transcripts also include a number coding for receptors expressed on neutrophils, including CXCR2.

**Table I.** Neutrophil genes with those upregulated in both resting and inflamed CCR1/2+ve monocytes marked with an asterisk

F3
MGAM*
PROM1*
MMP9
DHRS9
PPP1R42
VEGFA
S100A9*
MRGPRA2A
1810006J02RIK
GM13371
S100A8*
GM44165
MREG
WFDC21
ABCA13*
IFNLR1
MS4A3
CLDN1
HSD17B1
DNMT3L
LIN28A*
ELANE*
MRGPRA2B
FCNB*
OLFML2B*
F730016J06RIK*
PTGS2OS2
MPO*
INPP5J
MOGAT2
LCN2*
GM38575
IL1F9*
4930438A08RIK*
CXCR2*
NCAM1*
SYNE1
GM17494
G0S2
IGHV1-41
TST*
PAX8
CLDN15*
TREM3
1700012B09RIK
GM15536
GM19040
MAPK13
E230014E18RIK
GM3942
KNTC1
TREM1
ICA1*
CD24A
GCA
1700020L24RIK
FPR1*
9530077CO*

### CCR1/2+ve monocytes represent a homogeneous cellular population

**FIGURE 2.**
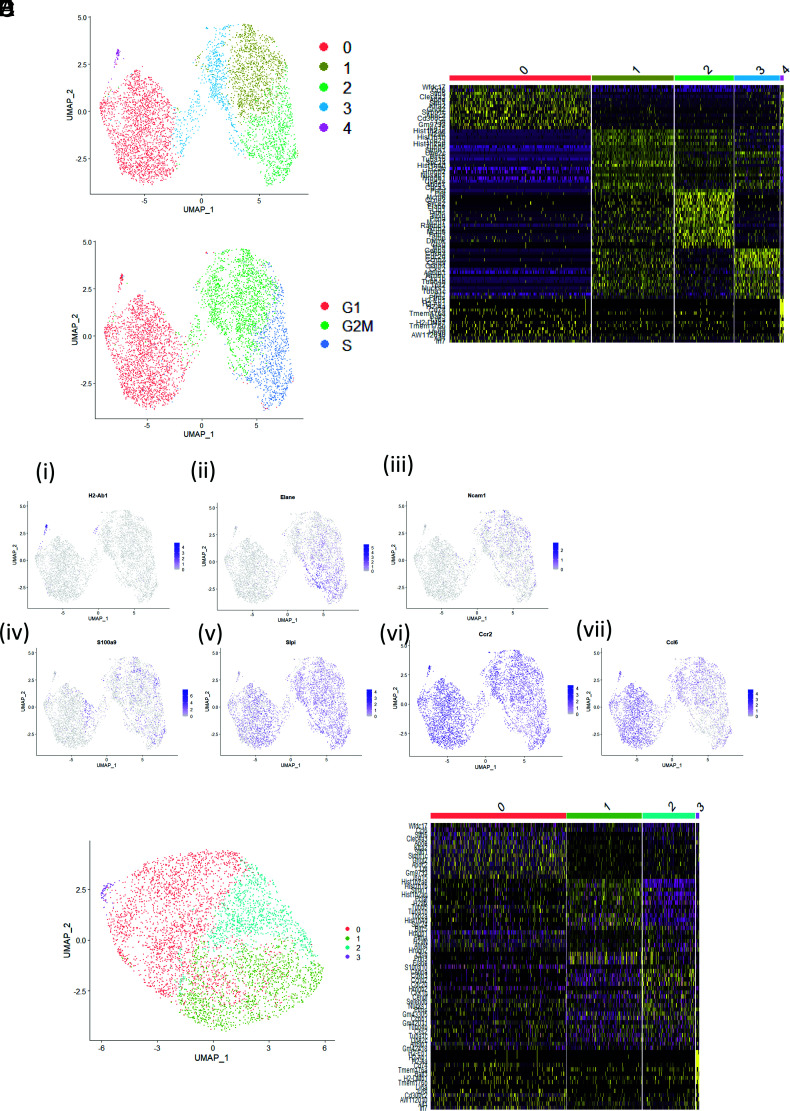
Single-cell sequencing analysis of CCR1/2+ve monocytes. (**A**) Two-dimensional UMAP visualization of bone marrow–derived CCR1/2+ve monocytes. Colors represent individual clusters of cells (0–4). Resolution = 0.21. (**B**) Heatmap depicting the top 20 differentially expressed genes within the CCR1/2+ve monocyte population that scaled during processing. (**C**) UMAP visualization of cell cycle analysis. (**D**) (i–vii) Feature plots depicting gene expression patterns of H2-Ab1, Elane, Ncam1, S100a9, Slpi, Ccr2, and Ccl6, respectively. (**E**) UMAP visualization of CCR1/2+ve monocytes with the effect of cell cycle regressed out of the data. (**F**) Heatmap depicting the top 20 genes that scaled during processing for each cluster after cell cycle regression.

The presence of transcripts, typical of neutrophils, in the CCR1/2+ve monocytes raised the possibility of neutrophil contamination. To address this, we carried out single-cell RNA-seq on sorted CCR1/2+ve monocytes. Data were visualized in a UMAP plot, and five distinct clusters were identified on the basis of differential gene expression patterns (Fig. 2A). A heatmap, showing the top 20 genes that scaled during processing for each cluster, is presented in Fig. 2B. Because several genes, identified during clustering, are associated with the cell cycle, cell cycle analysis was applied to the data, confirming that cell cycle position could largely explain differences observed between clusters; cells in cluster 2 were in S phase, cells in clusters 1 and 3 were in G_2_M phase, and cells in clusters 0 and 4 were in G_1_ phase (Fig. 2C). The expression of key differentially expressed genes is depicted as feature plots in Fig. 2D. Cluster 4 was identified as dendritic cells, based on high expression of H2-Ab1 (Fig. 2Di) in addition to other dendritic cell markers (data not shown). Neutrophil-associated genes Elane, Ncam1, and S100a9 were expressed by cells in either S or G_2_M phase of the cell cycle but absent from cells in G_1_ phase (Fig. 2Dii–2Div). Interestingly, expression of Slpi, the negative regulator of Elane, was high across all cells, regardless of cell cycle position (Fig. 2Dv). Similarly, expression of Ccr2 was high across all cells (Fig. 2Dvi). Expression of the chemokine Ccl6 increased as the cells leave G_2_M phase and enter into G_1_ phase (Fig. 2Dvii). Filtering out transcripts involved in the cell cycle significantly reduced heterogeneity within the data and revealed three clusters of CCR1/2 monocytes (plus cluster 3, which comprised dendritic cells) (Fig. 2E). Differential gene expression analysis between the clusters revealed that, after cell cycle regression, the top 20 genes expressed in clusters 1 and 2 remained largely associated with the cell cycle, suggesting there is little else contributing to variance within the data (Fig. 2F). CCR1/2+ve monocytes therefore represent a monodisperse population. No neutrophils were found within the population in this single-cell sequencing analysis, thereby ruling out neutrophil contamination as a contributor to the bulk RNA-seq data.

Thus, CCR2+ve and CCR1/2+ve monocytes both display a core monocytic gene signature, but the CCR1/2+ve monocytes are distinguished by expression of an additional neutrophilic gene expression profile. The transcription of genes such as MPO and ELANE in the CCR1/2+ve monocytes, which are not expressed in mature neutrophils (21), is a further indication that these cells are relatively undifferentiated.

### The CCR1/2+ve monocytic population is expanded in inflammation

**FIGURE 3.**
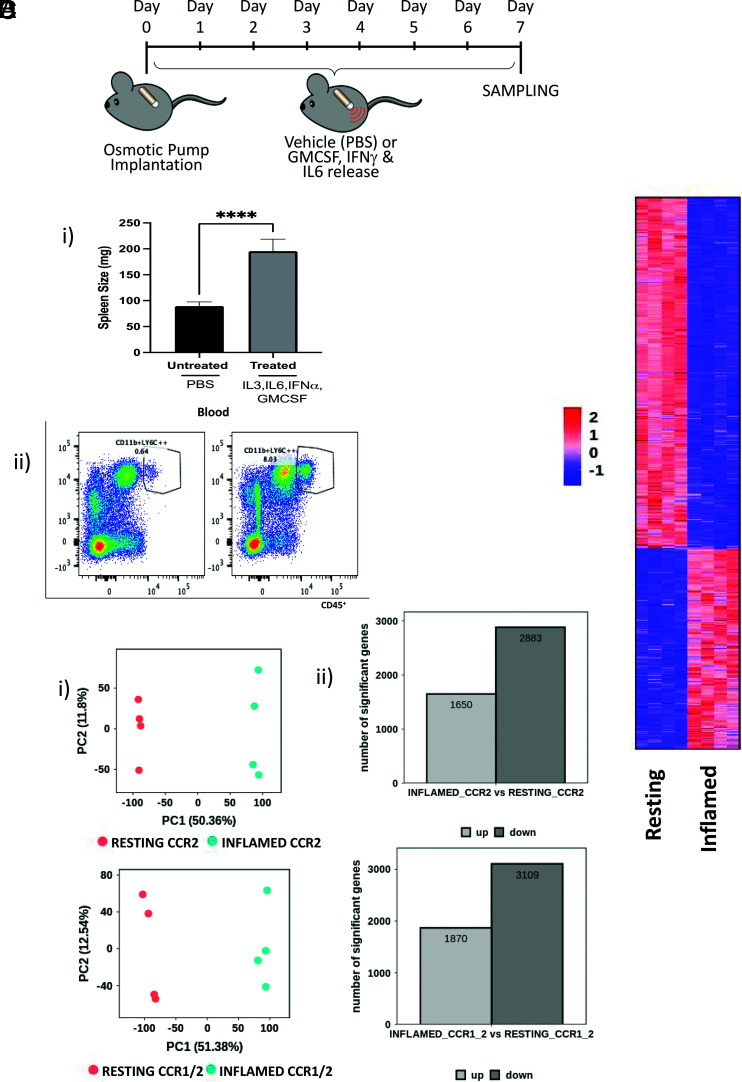
CCR2+ve and CCR1/2+ve monocytes are similarly affected by inflammation. (**A**) Diagram showing the experimental setup with vehicle, or cytokine-loaded, osmotic minipumps introduced at day 0 and tissue harvesting at day 7. (**B**) (i) Spleen weights (mg) in vehicle- and cytokine-treated mice. (ii) Expansion of monocytes in the cytokine-treated mice as shown in the righthand flow cytometry profile. (**C**) (i) Principal component analysis and (ii) number of the differentially expressed genes, comparing resting and inflamed CCR2+ve monocytes (upper panels) and resting and inflamed CCR1/2+ monocytes (lower panels). (**D**) Heatmap comparing resting and inflamed CCR1/2+ve monocytes. ****p < 0.0001.

We have previously demonstrated that, under inflammatory conditions, the percentage of CCR1/2+ve monocytes is increased in bone marrow and blood (13), although whether this relates to CCR2+ve monocytes now expressing CCR1 or specific expansion of the CCR1/+ve population is not clear. To test this, we subjected mice to prolonged cytokine-driven systemic inflammation (Fig. 3A), establishment of which was reflected in splenomegaly (Figure 3Bi) and a dramatic increase in monocytic cells in peripheral blood (Fig. 3Bii). Flow cytometric analysis indicated a clear increase in the numbers of CCR1/2+ve monocytes, as well as the mean fluorescence intensity for the CCR1 reporter on these cells, in both bone marrow and peripheral blood (Supplemental Fig. 3A, 3B).

Again, CCR2+ve and CCR1/2+ve monocytes were sorted, and bulk RNA-seq was performed. Direct comparison of the transcriptomes of resting and inflamed CCR2+ve and CCR1/2+ve monocytes revealed major shifts in gene expression following inflammation, as shown by principal component analysis (Fig. 3Ci). There were ∼2000 upregulated and 3000 downregulated transcripts separating CCR2+ve and CCR1/2+ve resting and inflamed monocytes, and essentially the same transcriptional differences are seen in both populations following inflammation (Fig. 3Cii, 3D). The top 50 upregulated transcripts in the inflamed populations, compared with their uninflamed counterparts, are listed in Table II. Gene ontology analysis indicates that the bulk of upregulated transcripts encode genes involved in inflammatory and antiviral responses, whereas those downregulated included genes involved in dendritic cell differentiation and cellular signaling (Supplemental Fig. 4A, 4B). Overall, these results indicate that inflammation induces essentially identical alterations in the transcriptomes of CCR2+ve and CCR1/2+ve monocytes.

**Table II.** Top 50 upregulated genes in inflamed CCR2 monocytes (essentially the same as CCR1/2 monocytes)

F11R
KCNAB2
SKIV2L
MROH1
MORF4L1
IL4RA
C4B
TRAPPC9
BAK1
ATP11A
HNRNPA3
MYH9
KDM5C
U2AF2
EMC1
GTF3C1
CARD9
ZC3H3
AP1S1
SLC52A3
KLHL18
GM3608
AI506816
ARID5A
GM5830
GCN1L1
SLC38A10
CD300LF
HCFC1
PYGB
DEF8
TMEM104
CAP1
EMILIN1
NUP188
DGLUCY
PIK3CD
UBA1
PRR14L
SUPT5
ARHGAP30
SZRD1
ZBTB17
LMF2
HYOU1
MDC1
SEC16A
TSC2
ENO1
PCSK7

### The inflamed CCR2+ve and CCR1/2+ve monocytes are transcriptionally distinct

**FIGURE 4.**
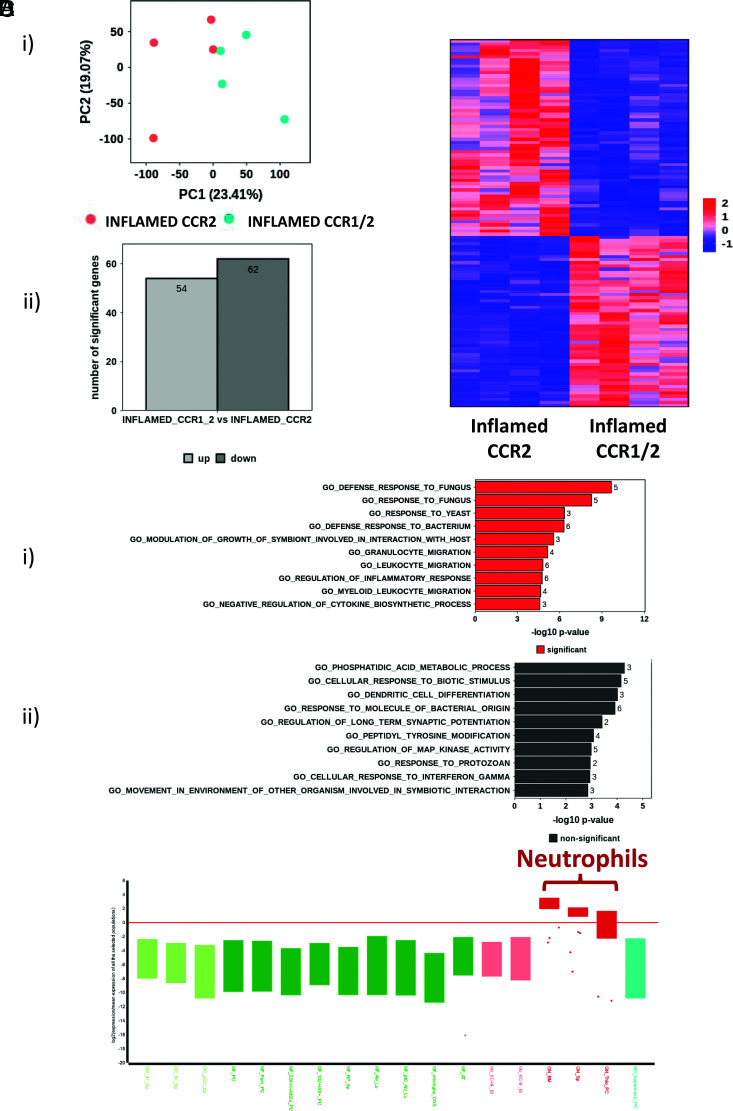
Inflamed CCR2+ve and CCR1/2+ve monocytes are transcriptionally distinct. (**A**) (i) Bulk RNA-seq principal component analysis of CCR1/2+ve monocytes and CCR2+ve monocytes. (ii) Bar graph showing the number of up- and downregulated genes expressed by CCR1/2+ve monocytes versus CCR2+ve monocytes. (**B**) Heatmap demonstrating distinct gene expression patterns between CCR1/2+ve monocytes and CCR2+ve monocytes. (**C**) Gene ontology analysis of transcripts (i) upregulated and (ii) downregulated in CCR1/2+ve monocytes compared with CCR2+ve monocytes. (**D**) Box plot showing maintenance of the neutrophilic gene expression pattern in inflamed CCR1/2+ve monocytes. This was obtained by entering the top 200 genes, preferentially expressed in inflamed CCR1/2+ve monocytes, into the engine server using the MyGeneset program, which analyzes transcript expression across immune and stromal cell populations.

We next compared the transcriptomes of the inflamed CCR2+ve and CCR1/2+ve monocytes. Principal component analysis (Fig. 4Ai) indicated that these two populations are related but distinct, with ∼60 upregulated and 60 downregulated genes separating the populations (Fig. 4Aii, 4B). Gene ontology indicated that upregulated transcripts in CCR1/2+ve monocytes correspond to response to pathogens and granulocyte migration, whereas downregulated transcripts display a mixed gene ontology profile (Fig. 4Ci, 4Cii). As with resting cells, the transcripts differentially expressed in the CCR1/2+ve monocytes preferentially align with neutrophilic transcriptomes (Fig. 4D), and ∼60% of the upregulated genes are the same as those seen upregulated in resting CCR1/2+ve monocytes (Table I). Thus, the core neutrophilic difference between CCR2+ve and CCR1/2+ve monocytes is maintained, indicating that the increased number of CCR1/2+ve monocytes is a result of expansion of the resting CCR1/2+ve population and not of de novo CCR1 expression on CCR2+ve monocytes.

### CCR1/2+ve monocytes are less able to differentiate to macrophages than CCR2+ve monocytes

**FIGURE 5.**
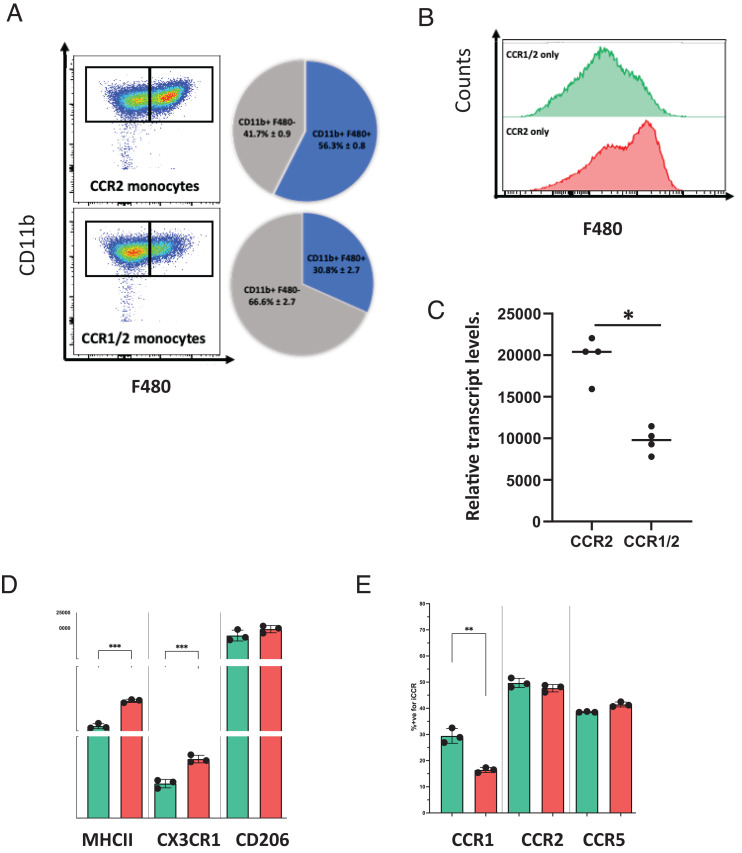
CCR1/2+ve monocytes are less able to differentiate to macrophages than their CCR2+ve counterparts. (**A**) Pie charts and associated representative FACS plots showing the proportion of fully differentiated CD11b^+^F480^+^ macrophages after 5 d of culture with CSF-1, starting from sorted bone marrow inflammatory monocytes (CCR2+ve and CCR1/2+ve coexpressing). (**B**) Representative histograms showing F480 expression of CCR2+ve (red) and CCR1/2+ve (green) monocytes cultured for 5 d with CSF-1. (**C**) CSF-1R expression, determined by RNA-seq, on CCR2+ve and CCR1/2+ve coexpressing sorted inflammatory monocytes. The Mann–Whitney *U* test was performed to determine statistical significance, with a *p* value of 0.05 defined as significant. (**D**) Expression of macrophage markers MHC class II, CD206, and CX3CR1 on the CD11b^+^F480^+^ and CD11b^+^F480^−^ fractions of cultures originating from sorted CCR1/2+ve monocytes (green) and sorted CCR2+ve monocytes (red) at day 5, expressed as mean fluorescence intensity. Each data point represents two mice. An unpaired Student *t* test was performed to determine statistical significance, with a *p* value of 0.05 defined as significant. (**E**) Expression of iCCRs (CCR1, CCR2, and CCR5) on the CD11b^+^F480^+^ and CD11b^+^F480^−^ fractions of cultures originating from sorted CCR1/2+ve monocytes (green) and sorted CCR2+ve monocytes (red) at day 5, expressed as mean fluorescence intensity of reporter proteins. CCR2 = Pe-Texas Red = mRuby2, CCR1 = FITC = Clover, and CCR5 = APC = IRFP682. Each data point represents two mice. An unpaired Student *t* test was performed to determine statistical significance, with a *p* value of 0.05 defined as significant. *p <* *0.05, **p <* *0.01, ***p <* *0.001.

To examine phenotypic differences between CCR1/2+ve and CCR2+ve monocytes, we imaged them using ImageStream, which showed (Supplemental Fig. 5) that these cells are broadly similar in shape with a suggestion of increased size in the CCR1/2+ve monocytes. Next, we investigated the relative abilities of CCR1/2+ve and CCR2+ve monocytes to differentiate in vitro to macrophages. As shown in Fig. 5A, 5B, CCR1/2+ve monocytes were less able to differentiate to F480+ve macrophages than CCR2+ monocytes. The limited differentiation seen with the CCR1/2+ve monocytes is in keeping with lower expression of CSF1R on the CCR1/2+ve monocytes (Fig. 5C). In addition, when comparing F480+ve macrophages differentiated from these two monocytic subtypes, those differentiated from CCR1/2+ve monocytes displayed reduced levels of MHC class II and CX3CR1 but equivalent levels of CD206 to the progeny of CCR2+ve monocytes (Fig. 5D). In terms of the relative expression of CCR1, 2, and 5 in the progeny (assessed using cells from REP mice), although levels of CCR2 and CCR5 were equivalent, the CCR1/2+ve monocytes gave rise to differentiated progeny displaying more extensive CCR1 expression (Fig. 5E). Thus, these data indicate that CCR2+ve and CCR1/2+ve monocytes display differences in their macrophage differentiation.

### CCR1 does not regulate CCR1/2+ve monocyte mobilization from the bone marrow or accumulation at inflamed sites

**FIGURE 6.**
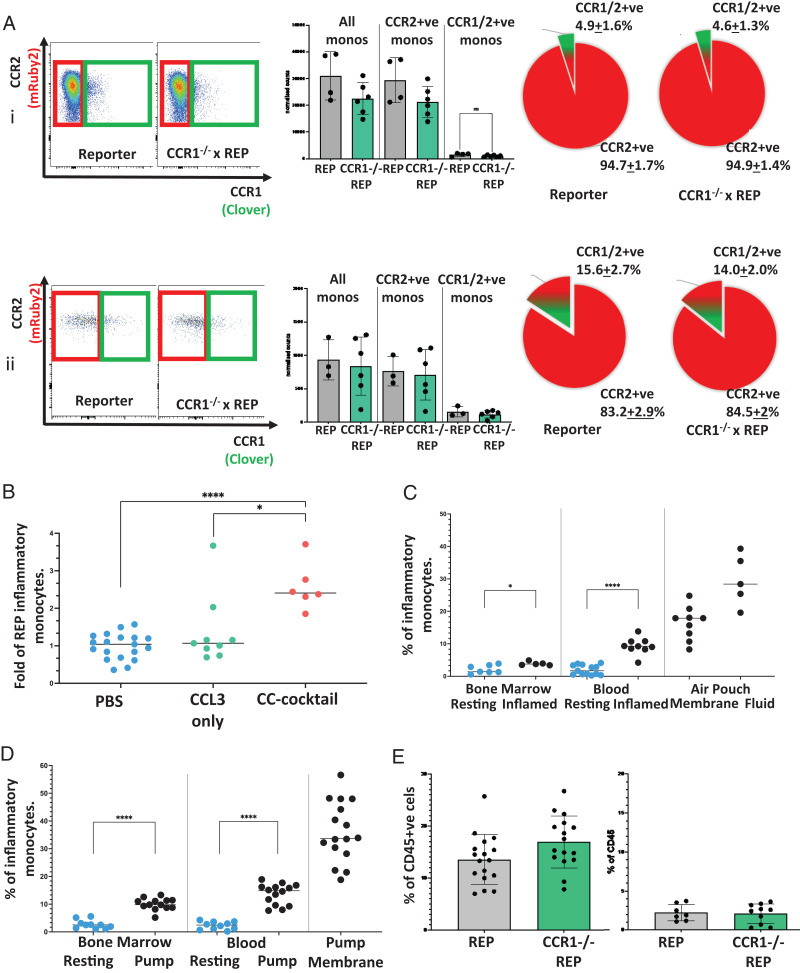
CCR1 does not contribute to the egress of the CCR1/2+ve monocytes from the bone marrow or their accumulation at inflamed sites. (**A**) (i) Representative FACS plots showing CCR2+ve (red box) and CCR1/2+ve (green box) inflammatory monocytes (CD11b^+^ Ly6C^++^ Ly6G^−^) in the bone marrow of REP and CCR1^−/−^ xReporter resting mice, with associated bar graph showing normalized monocyte counts and pie charts showing the proportion of CCR1/2+ve monocytes (green/red) of total CCR2+ve bone marrow monocytes (red). (ii) Representative FACS plots showing circulating CCR2+ve (red box) and CCR1/2+ve coexpressing (green box) inflammatory monocytes (CD11b^+^ Ly6C^++^ Ly6G^−^) from the blood of REP and CCR1^−/−^ xReporter resting mice, with associated bar graph showing normalized monocyte counts and pie charts showing the proportion of CCR1/2+ve monocytes (green/red) of total CCR2+ve circulating monocytes (red). (**B**) Number of inflammatory monocytes in circulation at day 9 of the air pouch model. Mice were injected 12 h before cull with chemokines to enhance monocyte extravasation. Numbers are expressed as the fold of PBS injection (blue), with two different chemokine injections, CCL3 only (green), and a CC chemokine mix containing CCL7, CCL3, CCL5, and CXCL5 (red). One-way ANOVA with Brown-Forsythe and Welch correction was performed to determine statistical significance, with a *p* value of 0.05 defined as significant. (**C**) Proportion of CCR1/2+ve monocytes (of total CD11b^+^Ly6C^++^ Ly6G^−^ CCR2^+^ monocytes) in bone marrow, blood, and periphery (air pouch membrane and fluid) during inflammation (black) compared with CCR1/2+ve coexpressing monocytes found in the resting state (blue). An unpaired Student *t* test was performed to determine statistical significance, with a *p* value of 0.05 defined as significant. *p <* *0.05. (**D**) Proportion of CCR1/2+ve coexpressing monocytes (of total CD11b^+^ Ly6C^++^ Ly6G^−^ CCR2+ve monocytes) in bone marrow, blood, and periphery (minipump membrane) during a slow cytokine release model of inflammation (GM-CSF, IL-3, IL-6, and IFN-α) compared with CCR1/2+ve monocytes found in the resting state (blue). An unpaired Student *t* test was performed to determine statistical significance, with a *p* value of 0.05 defined as significant. (**E**) Proportion of inflammatory monocytes (as a percentage of CD45^+^) in the membrane and fluid of inflamed REP and CCR1xReporter mice (air pouch model). ****p <* *0.0001.

Because CCR2 is essential for monocyte mobilization from the bone marrow to peripheral blood (12, 22–24), we next examined the involvement of CCR1 in CCR1/2+ve monocyte mobilization. To this end, we crossed REP mice with CCR1^−/−^ mice and examined the number of CCR1/2+ve monocytes in bone marrow and peripheral blood. No differences in CCR1/2+ve monocyte numbers were seen in bone marrow or peripheral blood in the absence of CCR1 (Fig. 6A). Thus, CCR1 is not involved in the basal mobilization of CCR1/2+ve monocytes from bone marrow. To examine the possibility that CCR1 ligands, draining from inflamed sites in peripheral blood, specifically mobilize CCR1/2+ve monocytes from the bone marrow during inflammatory responses, we i.v. injected either the CCR1 ligand CCL3 or a mixture of CC chemokines, including ligands for CCR2. As shown (Fig. 6B), CCL3 did not mobilize CCR1/2+ve monocytes, whereas, as expected, administration of the chemokine mixture did. Overall, therefore, these data indicate that CCR1 is not involved in CCR1/2+ve monocyte mobilization under either resting or inflamed conditions.

Next, we examined the possibility that CCR1/2+ve monocytes are preferentially recruited to inflamed sites. Analysis of CCR1/2+ve monocytes in the inflamed air pouch model (12, 25) (Fig. 6C) indicated that, as reported previously (13), the percentage of these cells (as a percentage of total inflammatory monocytes) increased in peripheral blood in response to air pouch inflammation. Furthermore, we observed a dramatic increase in the percentage of these cells in the inflamed air pouch, where they comprised ∼26% of total inflammatory monocytes. Similarly, when we studied mice implanted with the cytokine-loaded slow-release capsules to induce peripheral inflammation (Fig. 6D), we again saw an increase in the percentage of CCR1/2+ve monocytes in the blood of inflamed mice and a marked increase (>30% of total inflammatory monocytes) in the tissue surrounding the implanted pump. Crucially, we also saw accumulation of CCR1/2+ve monocytes in the inflamed air pouch in REP mice with a homozygous deletion in CCR1 (Fig. 6E), indicating that CCR1 does not account for the preferential recruitment of these cells to inflamed sites and that this may relate to the increased adhesion molecule expression in these cells resulting in enhanced CCR2-activated adhesion to the luminal endothelium. (Table III). Overall, these data indicate that CCR1/2+ve monocytes cannot use CCR1 for mobilization from the bone marrow and accumulate at inflamed sites in a CCR1-independent manner.

**Table III.** Relative expression of adhesion molecules in CCR1/2+ve compared with CCR2+ve monocytes

Gene name	Log_2_ fold change	Adjusted *p* value
VCAM1	4.06	3.81E-10
ITGAD	5.7	0.000103361
NCAM1	1.68	9.84E-22

## Discussion

There has been much interest in the issue of redundancy of function of chemokines and their receptors, particularly in the context of inflammation. We have been studying this with respect to the receptors CCR1, CCR2, CCR3, and CCR5, which sit within a tightly integrated chromosomal locus in the mammalian genome and which regulate nonneutrophilic myeloid cell recruitment to inflamed sites. In this study, we demonstrate that although the majority of monocytes express only CCR2 from this locus, a subset of monocytes coexpress CCR2 and CCR1. These cells are present at similar levels in bone marrow, blood, and spleen, and their expression of CCR1 is elevated upon induction of systemic inflammation. Given the similar levels of CCR1/2+ve cells in bone marrow and blood, it is our assumption that they originate from the bone marrow. Our analyses highlight clear differences between the CCR2+ve and CCR1/2+ve monocyte populations, and this is further reinforced by RNA-seq, which reveals them to be transcriptionally distinct. Importantly, these data demonstrate that CCR1/2+ve monocytes are not simply CCR2+ve monocytes with stochastic CCR1 expression but a transcriptionally distinct monocyte subpopulation.

We have previously demonstrated that the majority of monocyte recruitment to inflamed sites is nonredundantly dependent on CCR2 (12). Our previous analyses indicated that the small number of monocytes that do enter tissues in a CCR2-independent manner are transcriptionally distinct from the CCR1/2+ve monocytes reported in this study and therefore that monocytes with a neutrophilic gene signature are excluded from inflamed sites in CCR2^−/−^ mice. Overall, this indicates that the CCR1/2+ve cells require CCR2 to enter inflamed tissues. This conclusion is reinforced by data from REP mice lacking CCR1, which indicate that CCR1 plays no role in CCR1/2+ve monocyte recruitment to inflamed sites. CCR1 also is not involved in the mobilization of these cells from the bone marrow. Our conclusion therefore is that these cells most likely require CCR1 for migration within a tissue only after having extravasated from the vasculature. It is possible that the increased accumulation at inflamed sites reflects the increased expression of adhesion molecules in the CCR1/2+ve monocytes.

Transcriptionally, what most distinguishes CCR1/2+ve monocytes from their CCR2+ve counterparts is expression of a sizable cohort of neutrophil-specific genes. Further single-cell analysis indicates that the CCR1/2+ve cells represent a monodisperse population of monocytes and that the neutrophil-specific transcripts are not a result of neutrophil contamination during cell sorting. Interestingly, although typical monocyte-related genes within the CCR1/2+ve population are expressed in cells at all stages of the cell cycle, the neutrophil-specific genes are typically not seen in G_1_ phase but are seen to be expressed in all other phases of the cell cycle. It is possible that this represents an immediate early response (26) in this monocyte population, which is triggered as they enter the cell cycle, and that quiescent cells, or cells in G_1_, do not express the neutrophil-associated genes.

Intriguingly, there have been numerous previous reports of atypical monocyte populations characterized by expression of neutrophil-specific genes (27–31) and generally assessed as having immunosuppressive activities (27, 29, 30). Our data particularly align with the cellular population reported by Yáñez et al. (31), who demonstrated that these cells arise from granulocyte-macrophage progenitor cells without going through the classic myeloid dendritic precursor stage. Despite the strong transcriptional similarity to monocytes, these cells are therefore produced independently of monocyte–dendritic cell precursors.

Overall, our data demonstrate that monocytes can express alternative combinations of CCR1 and CCR2. However, close examination shows that CCR1/2 expression delineates a functionally and transcriptionally independent population. Our data therefore reveal specificity, rather than redundancy, in iCCR expression by monocytes.

## Supplementary Material

Supplemental 1 (PDF)
