# Supplementary material for: CCR1 and CCR2 Coexpression on Monocytes Is Nonredundant and Delineates a Distinct Monocyte Subpopulation
Source: J Immunol. 2024 Jun 3;213(2):214–25. doi: 10.4049/jimmunol.2400007 (PMC11215633; doi:10.4049/jimmunol.2400007)
Supplement: Supplemental 1 (PDF) [file JI_2400007_Supplemental_1.pdf]

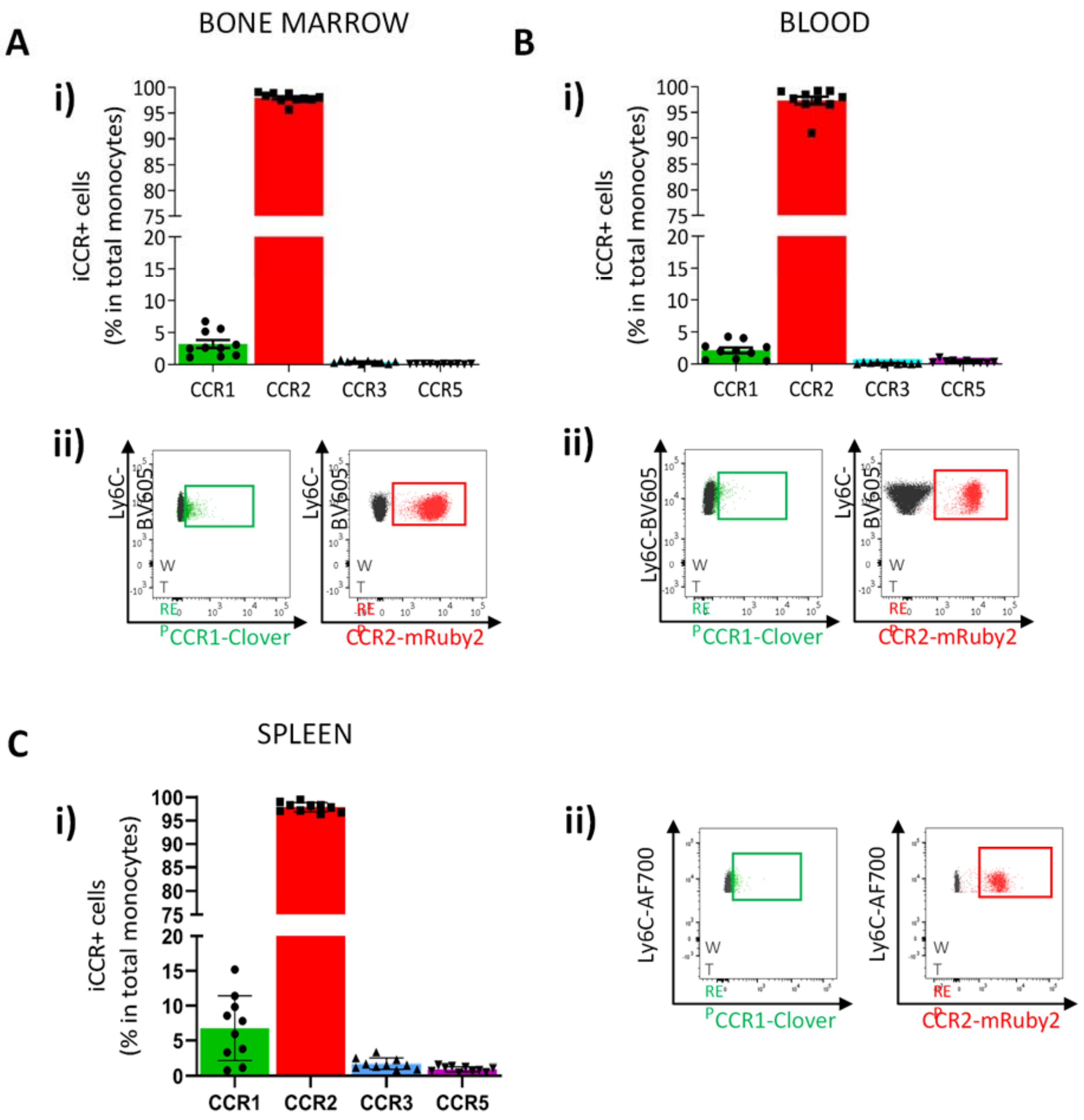

Supplementary Figure 1: Results from flow cytometric assessment of CCR1 and CCR2 expression on monocytes in bone marrow (A), blood (B) and spleen (C). Data are represented as bar charts (i) and flow cytometry profiles (ii).

A

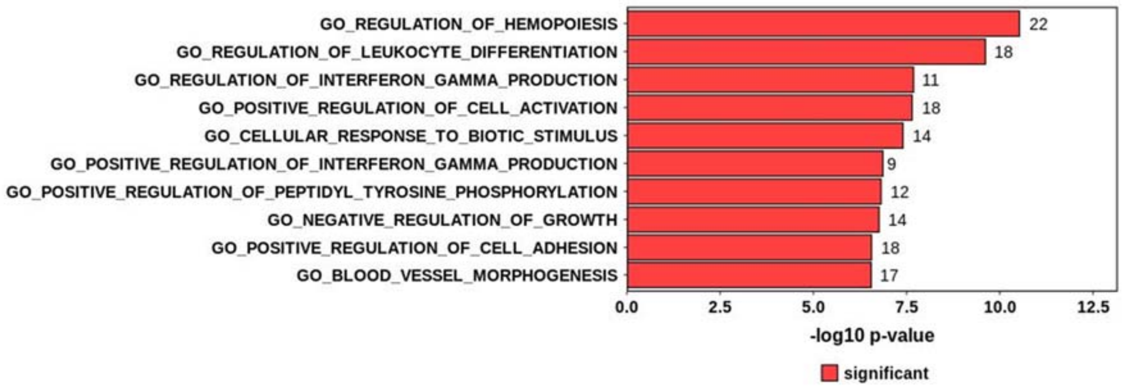

B

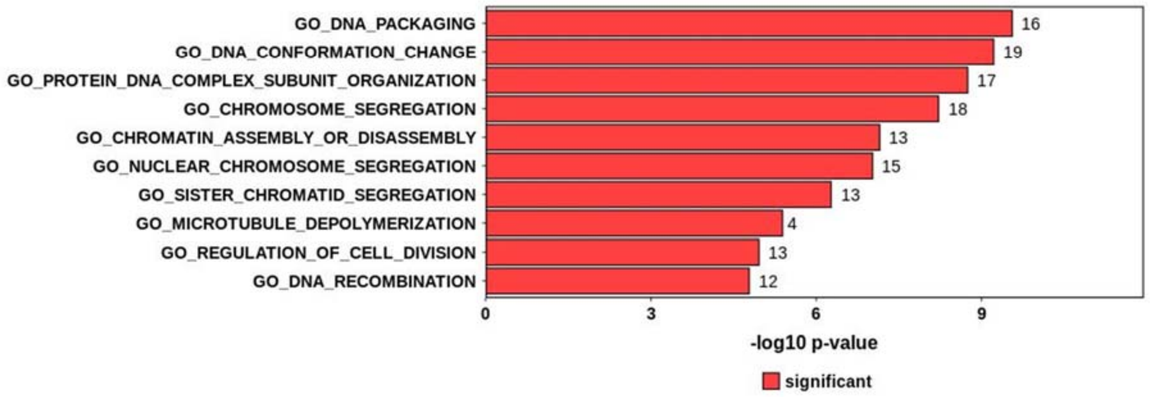

Supplementary Figure 2: Gene ontology analysis of transcripts upregulated in A) CCR2+ve monocytes; B) CCR1/2+ve monocytes.

A

B

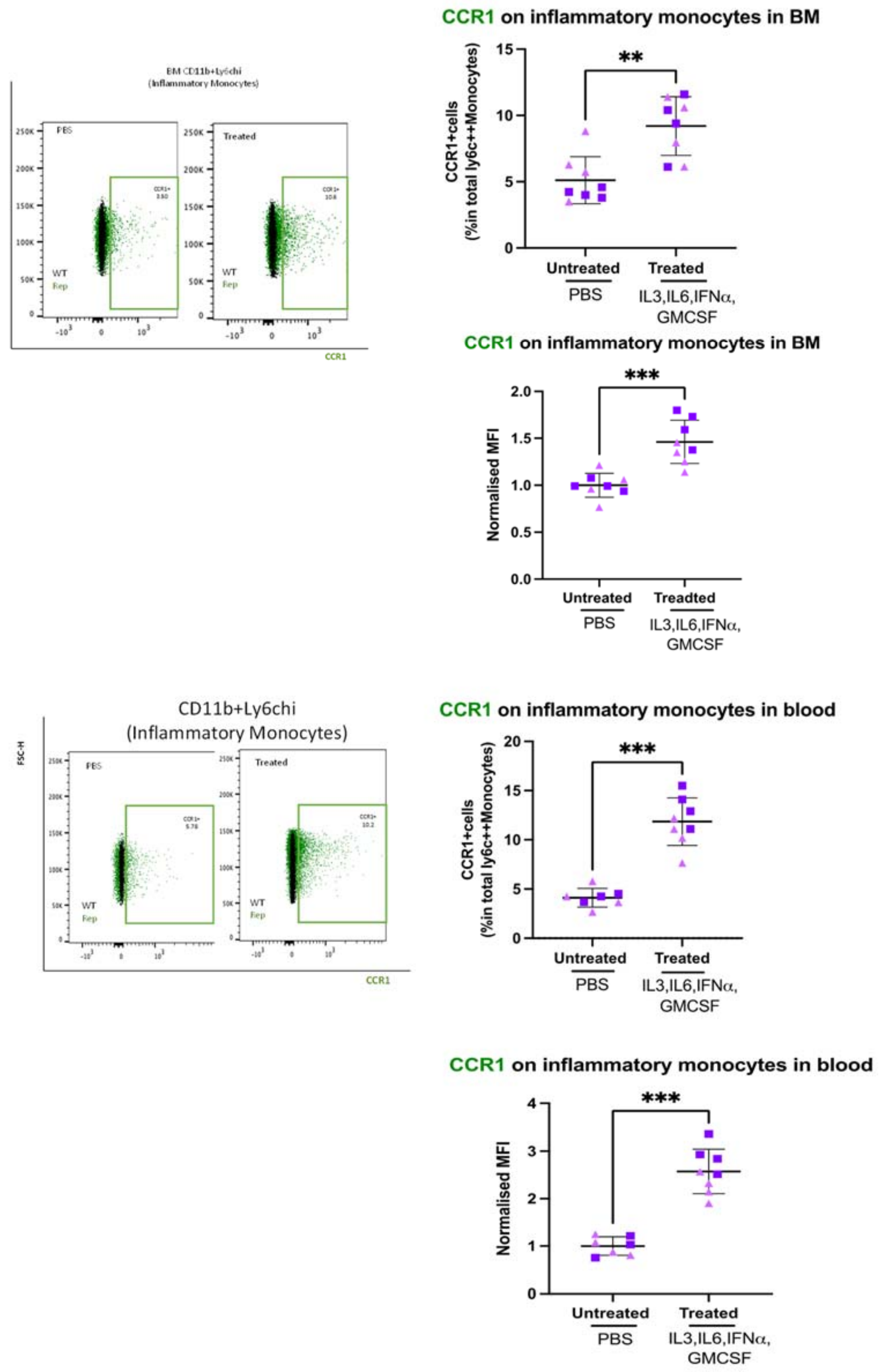

Supplementary Figure 3: Flow cytometric data showing the numbers of cells expressing ccr1 and the level of expression in CCR1/2+ve monocytes in A) bone marrow and B) peripheral blood.

A

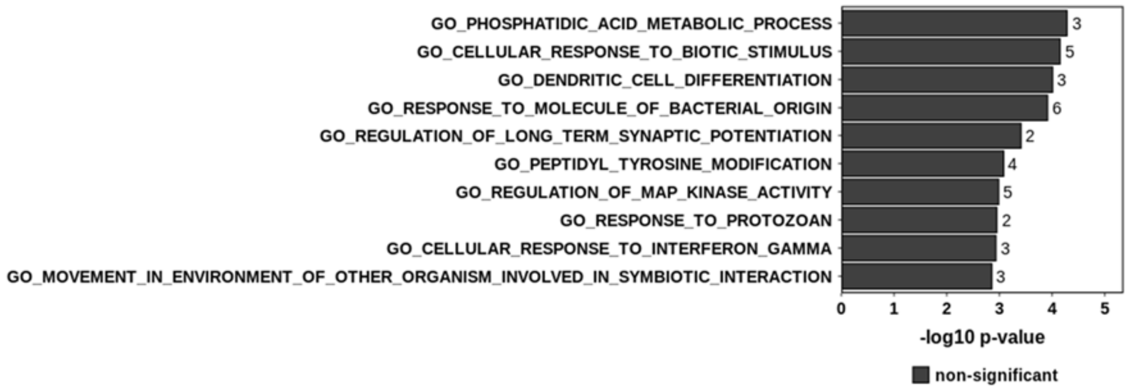

B

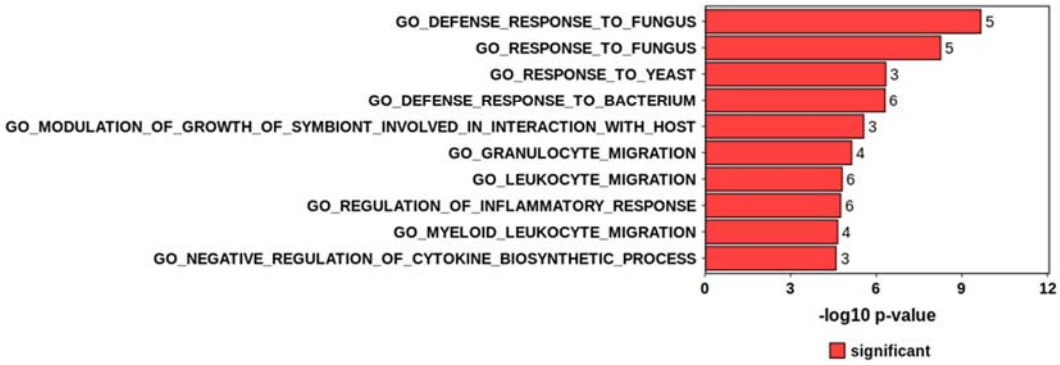

Supplementary Figure 4: Gene ontology analysis of transcripts upregulated (A) and down regulated (B) in inflamed CCR2+ve and CCR1/2+ve monocytes.

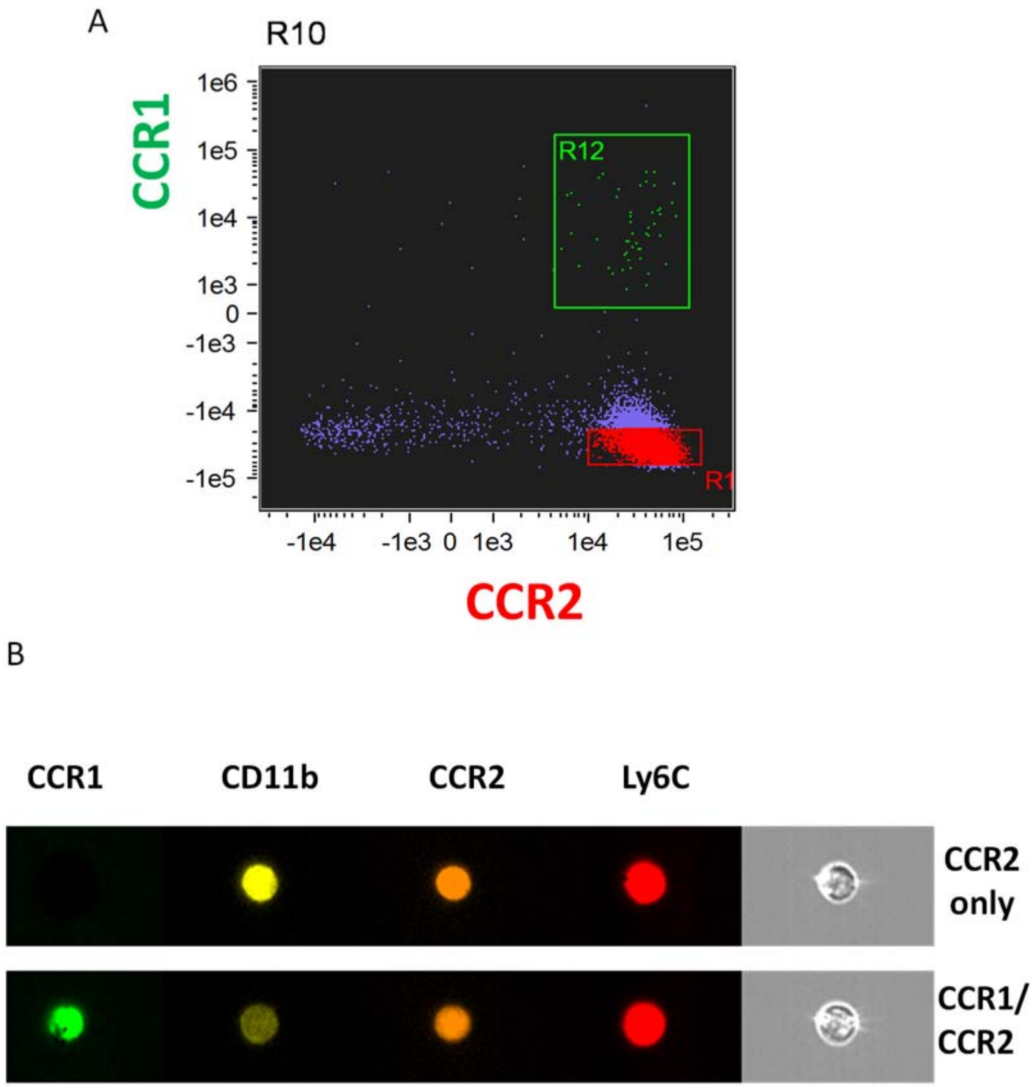

Supplementary Figure 5: A) Representative ImageStream panels showing the gating strategy for analysing CCR2+ve (Red Box) and CCR1/2+ve bone marrow inflammatory monocytes (Green box) for analysis of surface marker expression. B) Representative ImageStream panels comparing of CCR1, CD11b, CCR2 and Ly6C expression of CCR2+ve (top row) and CCR1/2+ve bone marrow monocytes. Note that the variability in CD11b fluorescence is not quantitative, but a consequence of the selected images. In addition, the apparently lower area occupied by the CCR1 and CCR2 reporters relates to their exclusively cytoplasmic localisation.

Supplementary Table 1.

Antibody panel – Flow cytometry analysis of BM and Pump membrane (on BD LSRFortessa)

- Live/Dead – e506
- CD45 – PerCP-Cy5.5
- Ly6C – AF700
- CD11b – APC-Cy7
- MHCII-BV605
- SiglecF – BV711
- F480 – BV786
- CD19 – PE-Cy7
- Ly6G – BUV395
- CD11c – BUV737

Supplementary Table 2.

Antibody panel – Monocyte sorting from BM (on FACS Aria)

- Live/Dead – e506
- CD45 – PerCP-Cy5.5
- CD11b – APC-Cy7
- Ly6C – BV605
- Ly6G – AF700
- SiglecF – AF700
- CD19 – AF700
